# Measuring psychological distress using the 12‐item general health questionnaire and the six‐item Kessler psychological distress scale. Psychometric comparison and equipercentile equating of the two scales

**DOI:** 10.1002/mpr.2033

**Published:** 2024-07-04

**Authors:** Andreas Lundin, Joseph Junior Muwonge, Maria Lalouni, Johan Åhlén

**Affiliations:** ^1^ Centre for Epidemiology and Community Medicine Stockholm Sweden; ^2^ Department of Global Public Health Karolinska Institute Stockholm Sweden; ^3^ Department of Clinical Neuroscience Karolinska Institute Stockholm Sweden

**Keywords:** agreement, crosswalks, equating, mental distress

## Abstract

**Objectives:**

This study aimed to examine if the General Health Questionnaire (GHQ)‐12 and Kessler 6 (K6) assess the same underlying construct and to develop a score conversion table for the two scales.

**Methods:**

A random sample of 4303 people who completed both the GHQ‐12 and K6 in 2021 were analyzed. Exploratory bifactor analysis evaluated if both scales measured the same construct, and Rasch analysis assessed item severities. The scales were transformed using Equipercentile equivalence for comparability and score conversion. Agreement was estimated with Cohen's Kappa coefficient, along with raw positive and negative agreement.

**Results:**

We found that the two scales measure the same phenomenon to the extent that they can be made equivalent. Conversion tables between GHQ‐12 and K6 are presented. Applying the commonly used cut‐off of ≥3 on the GHQ‐12 bi‐modal scoring, we found that the best corresponding cut‐off on the K6 would be ≥8. The prevalence of psychological distress was then 22% with GHQ‐12% and 21% with K6.

**Conclusions:**

The GHQ‐12 and K6 measure the same construct and corresponding cut‐off scores on one scale were found for the other scale. This is valuable for longitudinal studies or time series where one scale has replaced the other scale.

## 1 INTRODUCTION

Mental health conditions are increasing worldwide and cause one in 6 years lived with disability according to the WHO (WHO, 2024). Short non‐specific screening scales serve an important role, both to detect mental health conditions in health care settings and as indicators of the overall burden in public health surveys. One of the most used scales is the General Health Questionnaire‐12 (GHQ‐12), comprising 12 items on affective and functional distress. The GHQ‐12 is a measure of psychological distress, not linked to any specific psychopathologic entity (Goldberg, 1972). The GHQ‐12 was developed from the larger General Health Questionnaire scale, by choosing 12 items that were common among patients with less severe disorders (anxiety, depression, and stress reactions) and discriminated the patient group from general population controls. During the last few years, a similar distress scale has gained in popularity ‐ the Kessler Psychological Distress Scale, in a six‐ or ten‐item form (K6 and K10; Kessler et al., 2002). These were originally developed for the US National Health Interview Survey (NHIS) and were later included in the WHO's World Mental Health Survey (Kessler et al., 2010). Similar to the GHQ‐12, the K6 and K10 were developed to be used in population surveys as global measures of distress. However, there were some notable differences in how they were developed. Item content was restricted to symptoms of depression and generalized anxiety and item selection was based on item response theory (IRT), which is a more modern approach used to maximize measurement precision.

Both the GHQ‐12 and the K6 were developed as screening instruments and global measures of distress in population surveys. Both assess complaints that are common, but not specific to any specific mental disorder, but they differ in length, in their response options, and to some extent content. It is therefore not completely obvious that the instruments are equivalent. The criterion validity, that is, the ability to accurately screen for mental health conditions, have been compared for the GHQ‐12 and the K6 (Cornelius et al., 2013; Furukawa et al., 2003; Patel et al., 2008). In two of the studies the K6 outperformed the GHQ‐12 (Cornelius et al., 2013; Furukawa et al., 2003) and in the third study the GHQ‐12 outperformed the K6. However, no study has yet assessed how scores on GHQ‐12 correspond to scores on K6. Such comparison may be very useful, not the least in repeated cross‐sectional surveys and in longitudinal studies where the K6 has replaced the GHQ‐12. Moreover, crosswalks can facilitate cross‐population comparisons, particularly in cases where different distress scales have been employed. Batterham and colleagues developed crosswalk tables between eight measures of distress in an Australian convenience sample, including K10 and K6, but did not include GHQ‐12 (Batterham et al., 2017). Recently Jongsma and colleagues used three well known British cohorts together with a new calibration sample to equate scores of the GHQ‐12 and five other distress scales, but not including K6 (Jongsma et al., 2023). This study therefore provides an important addition to these studies.

The aims of the present study were to examine if the GHQ‐12 and the K6 assess the same underlying construct, to assess which score on one scale corresponds to a specific score on the other, and to examine the level of agreement between the scales.

## 2 METHODS

### 2.1 Study sample

Using stratified random sampling, a total sample of 50,270 individuals aged 16 and older was drawn from 38 municipalities and city districts in Stockholm County in 2021. After excluding persons who were dead or had moved from Stockholm County the eligible sample was 47,855 individuals. The invitation to participate was sent together with a link to a web survey and was also followed by a postal survey. The questionnaires consisted of questions about health, lifestyle, risk factors, background information, and psychological distress. The main instrument used to measure psychological distress was the K6 and was part of the main questionnaire. A random sample of those invited to participate (20%) received an extended questionnaire which included the 12 items of the GHQ‐12 (*n* = 9558 in total). A total of 23,072 individuals participated in the survey, 48.2% of the total invited. The sample that answered the questionnaire comprising the GHQ‐12 was 4531 individuals (47.4% response rate). In this study, we excluded individuals with missing items on the GHQ‐12 or K6 instruments, resulting in an analytic sample of 4303 individuals.

### 2.2 Measures

#### 2.2.1 GHQ‐12

The GHQ‐12 was constructed based on questions about depression, anxiety, and social impairment, by selecting those that differed most in occurrence between a clinical population (primarily with neurosis) and a control without reported psychiatric problems (Goldberg, 1972). The instrument comprises of questions on how frequently six symptoms about feelings/behaviors and six symptoms related to functioning have occurred in the past few weeks. It has both positively and negatively worded items to correct for the tendency to generally answer positively or negatively. The negatively worded questions all have the same response options, namely, (1) Not at all, (2) no more than usual, (3) more than usual, and (4) much more than usual. The positively worded questions have the following response options, from “best health” to “worst health”, namely: (1) More than usual/better than usual, (2) as usual, (3) worse/less than usual, and (4) much worse/less than usual. The Swedish version of the GHQ‐12 was originally translated by Diderichsen and Janlert (1992) and the criterion validity has been examined elsewhere (Lundin et al., 2016, 2017).

Two types of summary scores are used on the GHQ‐12. Either the answers are scaled 0–3 which are summed (also called the Likert scoring, 0 1 2 3), or the individual questions are dichotomized (0–1) and the sum scores are added together (bi‐modal scoring, 0 0 1 1). The former can be said to be an intensity and the latter a symptom count. We used both scoring methods in this study.

#### 2.2.2 Kessler 6

The six‐item Kessler scale was developed and published in 2002 (Kessler et al., 2003) for the screening of symptoms alike the Diagnostic and Statistical Manual of Mental Disorders (DSM) symptoms for depression and generalized anxiety in the general population. The K6 has been used in several population surveys including the WHO's World Mental Health Survey (Kessler et al., 2010). The Swedish version used in both the Stockholm public health surveys, “Hälsa Stockholm”, and National public health survey, “Health on equal terms” is a translation by the Center for Epidemiology and Community Medicine (CES). Both K10 and K6 are free to use without permission (available here: https://www.hcp.med.harvard.edu/ncs/k6_scales.php).

The K6 comprises of questions that asks how much of the time in the past 30 days, a person has felt nervous, hopeless, restless, or fidgety, so depressed that nothing could cheer them up, that everything was an effort, and worthless. It has five responses: (1) None of the time, (2) a little of the time, (3) some of the time, (4) most of the time, and (5) all of the time with scoring of 0 1 2 3 4. The sum of scores is between 0 and 24.

### 2.3 Procedure and statistical analysis

As a first step of examining the possibility of linking the two scales, we examined Pearson and Spearman correlation between the scale scores. As a rule of thumb, correlations above 0.70 are considered a lower bound for linking (Fayers & Hays, 2014). We then proceeded to exploratory bifactor analysis to investigate to what extent the K6 and GHQ‐12 measure the same phenomenon/construct. Scree test from Principal Component Analysis (PCA), Horn's Parallel Test (Horn, 1965), Velicier's Minimum Average Partial Factor Retention Method, MAP (Velicer, 1976) and Revelle's Very Simple Structure, VSS (Revelle & Rocklin, 1979) were used to estimate the number of factors to extract. The bifactor analysis was used to investigate how much of the explained variance is common and how much of the variance measured is due to something unique. Further, we tested to what degree each question contributes to the common factor using bifactor analysis. Factor loadings and variance based on Omega Hierarchical (Omega H) and Explained Common Variance are presented to indicate the unidimensionality. While unidimensionality is relative, high values on Omega H and ECV indicate that from a measurement perspective there is a dominating general dimension in the data. Unidimensionality is not a requirement for linking, but in order to claim that the result can be used interchangeably ‐said to be equated‐the two scales must reflect the same phenomenon.

Next, we investigated differences in severity by examining the distribution of responses in the raw total scores, and the severity of each question's response options and differences in measurement goals. The distribution of raw scores reflects partially the distribution of phenomena studied and the design of the scale. Since the population remained constant, differences in distribution are likely to be due to the severity of the items. The Partial Credit Model (PCM), a Rasch model for polytomous questions, was used to investigate the relative severity of each question. This was done by examining the correlation between the probability of choosing a specific response option and a latent (modeled) measure of mental illness. Response option severity was used to construct information curves for the two scales to examine the scales target with regard to measurement precision.

Because the distributions of the two scales were different, we used equipercentile linking to equate the two tests (Kolen & Brennan, 2014), that is, transform values of one scale into another. This method determines the relative position of scores through percentiles—the transformed values are then adjusted to be the same as the raw scores in the same percentile group. The conversion tables between the GHQ‐12 and K6 thus aim to find which score on one instrument corresponds to a certain score on the other instrument, while correlation coefficients provide answers to the degree to which the two scales generally co‐vary. Equipercentile linking belongs to the traditional linking methods and was chosen over “modern” IRT linking methods because (1) it does not require the strong assumptions of IRT (e.g. choosing the correct statistical model and assumption concerning the distribution of true scores) and (2) with a single group design there was not any doubt of non‐equivalent groups, which is the principal requirement in traditional linking. With the scales equated, that is, put on the same metric, Bland‐Altman figures were used to examine the overall level of agreement. Lastly, since both GHQ‐12 and K6 are often used by dichotomization, we compared the agreement at different cut‐offs values that are commonly used. The measure of agreement used was Cohen's kappa coefficient, a common test that adjusts for chance (McHugh, 2012). We also calculated the unadjusted agreement (proportion of total agreement) and the percentages of positive and negative agreement, measures that have their counterpart in sensitivity and specificity.

## 3 RESULTS

### 3.1 Do GHQ‐12 and K6 measure the same phenomenon?

**FIGURE 1:**
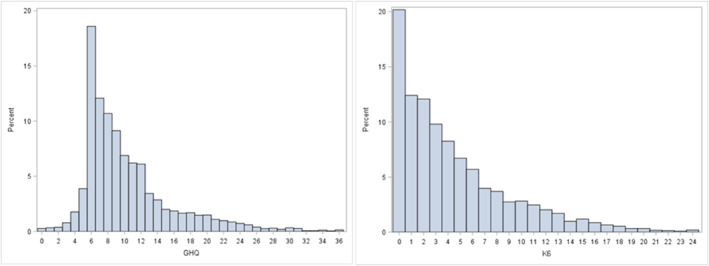
Proportional distribution of scores on the GHQ‐12 (left) and K6 (right). GHQ, general health questionnaire; K6, Kessler 6.

The Pearson and Spearman correlation coefficients were 0.77, meaning that 59% of the variance on one scale is explained/predicted by the other scale. The distribution of responses in the population (Figure 1) shows that both GHQ‐12 (left graph) and K6 (right graph) have asymmetric distributions, with a positive skew. The skew is more obvious in K6.

To further investigate possible covariation structures, several tests were performed on the GHQ‐12 and K6 jointly. The first three eigenvalues from a PCA of the items from both scales were: 8.75, 1.10 and 0.59, indicating a dominant factor and possibly, an additional one (based on eigenvalue >1). Horn's Parallel test, which adjusts for measurement error through simulations, also suggested two factors. The Revelle's Very Simple Structure test suggests a minimum of one factor and a maximum of two (complexity = 0.93 and 0.95, respectively). The Revelle's Very Simple Structure suggests a minimum of one factor and a maximum of two (complexity = 0.93 and 0.95, respectively).

Based on these test results, we proceeded on the basis that the GHQ‐12 and K6 contained a strong dominant component, but that there is possibly another covariation pattern in the data. Bifactor analysis was therefore performed to examine the unidimensionality and size of the general and two additional specific factors. The results are presented in Table 1.

**TABLE 1:** Factor loadings from bifactor analysis.

Scale	Item	General factor	Specific factor 1	Specific factor 2
K6	Nervous	0.64	0.54	
	Hopeless	0.72	0.52	
	Restless	0.53	0.41	
	Depressed	0.73	0.51	
	Fidgety	0.67	0.47	
	Worthless	0.71	0.50	
GHQ	Able concentrate	0.69		0.36
	Lost sleep	0.58	0.35	
	Play a useful part	0.59		0.44
	Make decisions	0.70		0.49
	Under strain	0.64	0.40	
	Face problems	0.73	0.36	
	Enjoy activities	0.68		0.46
	Overcome difficulties	0.68		0.45
	Depressed	0.77	0.38	
	Lost confidence	0.78	0.37	
	Felt worthless	0.76	0.38	
	Felt happy	0.64		0.33

*Note*: Fit indices RMSEA = 0.13, BIC = 12,935, compared with just a general factor with no group factors RMSEA = 0.18, BIC = 28,688.

**FIGURE 2:**
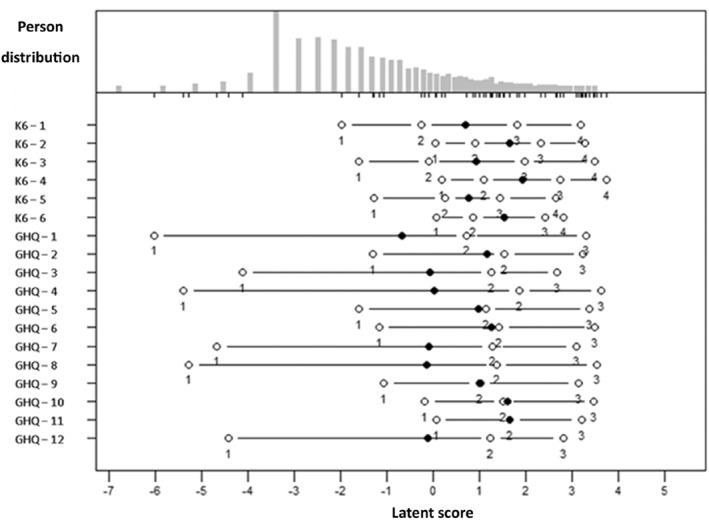
Item map of the severity of the response options.

The bifactor analysis clearly differentiated between the two scales, the specific factors consisted of questions from the respective scales. However, none of the questions had a strong loading on the specific factors. Omega Hierarchical was 0.77, which is interpreted as 77% of the variance in raw scores is due to the general factor. EVC, a measure of the common variance of the general factor was of similar strength (0.70). Most of the variance was thus explained by the general factor. The questions that contributed the most to the general factor (factor loadings) were as follows: for K6, questions 2 (hopelessness) and 4 (depressed); and for GHQ‐12, questions 9 (depressed), 10 (hopelessness), and 11 (uselessness). Notably, all these questions were about symptoms associated with depression. Given that the two scales were more similar than different (i.e., unidimensional), the next step was to investigate design effects from response options through fitting the PCM. Figure 2 shows a Wright map plotting the Thurstone thresholds of the K6 and GHQ‐12 item responses against the latent trait computed for the items. Each point represents where the transition (a 50/50 odds) to higher response alternatives occurs.

The first circle (on the left of the figure) shows where the second answer option in each question transitions to being more likely than the first, the second point where the third option is more common than others, and so on. It was evident that six of the GHQ‐12 questions had responses that were used by those with relatively low scores on the latent scale, which was a difference between the scales. These six questions were the GHQ‐12 questions that are positively worded.

Supplementary Figures S1 and S2 show, for the individual scales, the so‐called information curves, which indicate measurement reliability according to person location on the latent scale. Information (reliability) peaks around logit 4 for both scales indicating similar target (Figures 1 and 2). In Figure S3, where the positively worded questions from the GHQ‐12 are excluded, the information curve appears more like K6.

### 3.2 Equivalence between GHQ‐12 and K6 and their degree of similarity

**FIGURE 3:**
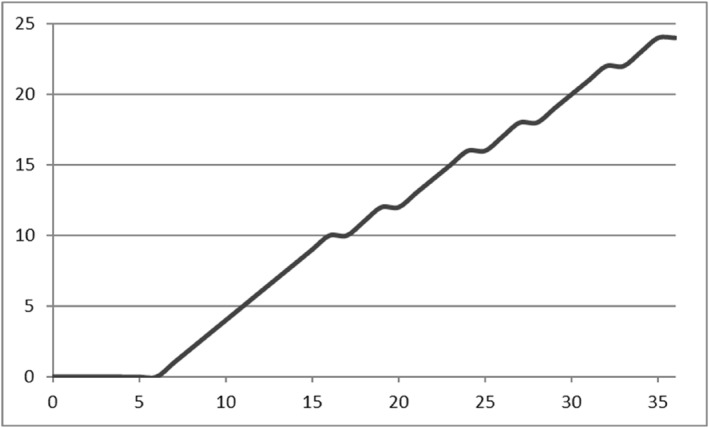
GHQ‐12 total scores (Likert, *x*‐axis) and corresponding K6 total score (*y*‐axis). GHQ, general health questionnaire; K6, Kessler 6.

Table 2 shows which scores on the K6 correspond to scores on the GHQ‐12 (Likert scoring) based on the equivalence. In addition, Table 3 shows the corresponding table for equivalence between K6 and GHQ‐12 (bi‐modal scoring). Figure 3 graphically shows the relationship between K6 and GHQ‐12 based on results shown in Table 2. The graph shows a non‐linear relationship. A K6 score of 13 (arbitrarily chosen) corresponds to 20.6 points on the GHQ‐12 (Likert scoring). For comparison, we used linear regression analyses to predict GHQ‐12 scores based on K6, and vice versa, to predict K6 scores based on GHQ‐12. We found that 13 points on the K6 corresponds to 18.0 points on the GHQ‐12, but 18 points on the GHQ‐12 corresponds to only 9.5 points on the K6.

**TABLE 2:** Conversion table between GHQ‐12 (Likert scoring, 0–36) and K6.

GHQ‐12 (Likert scoring)	K6
36	24
35	24
34	23
33	22
32	22
31	21
30	20
29	19
28	18
27	18
26	17
25	16
24	16
23	15
22	14
21	13
20	12
19	12
18	11
17	10
16	10
15	9
14	8
13	7
12	6
11	5
10	4
9	3
8	2
7	1
6	0
5	0
4	0
3	0
2	0
1	0
0	0

**TABLE 3:** Conversion table between GHQ‐12 (bi‐modal scoring, 0–12) and K6.

GHQ‐12 (bi‐modal scoring)	K6
12	24
12	23
12	22
12	21
12	20
12	19
12	18
11	17
11	16
10	15
9	14
8	13
7	12
6	11
5	10
4	9
3	8
2	7
2	6
1	5
0	4
0	3
0	2
0	1
0	0

Supplementary Figure S4 presents differences and similarity between K6 and the equivalent GHQ‐12 scores in a Bland‐Altman figure. On average, the difference between the transformed scores and the observed scores deviates by −0.27 points, indicating low bias.

#### 3.2.1 Agreement between cut‐off scores

Usually, specific cut‐off values are used to identify people with psychological distress on the GHQ‐12 (Likert and bi‐modal scoring) and K6. Table 4 shows the prevalence of the sample identified to have psychological distress and agreement between the scales based on commonly used cut‐off values and their corresponding score based on the conversion tables above. The prevalences are highly comparable, for instance, 22% of the sample scored three or more on the GHQ‐12 (bi‐modal scoring) and 21% were identified with the corresponding cut‐off value on K6 (8 or more). On the same cut‐off, positive agreement between GHQ‐12 (bi‐modal) and K6 is 66% and negative agreement about 90%. The corresponding agreement between K6 (8 or more) and GHQ‐12 cut‐off (14 or more on the Likert scoring) is slightly higher. See Table 4.

**TABLE 4:** Prevalence and agreement between cut‐off values between GHQ‐12 (Likert scoring, 0–36) or GHQ‐12 (bi‐modal scoring, 0–12) and K6 (0–24).

GHQ‐12 (likert) cut‐off values	GHQ‐12 prevalence	K6 cut‐off values	K6 prevalence	Kappa coefficient	Positive agreement	Negative agreement
≥12	29.00	≥6	30.54	0.63	0.74	0.89
≥13	22.89	≥7	24.84	0.63	0.71	0.91
≥14	19.45	≥8	20.87	0.62	0.69	0.92
≥15	16.59	≥9	17.17	0.59	0.66	0.93
≥16	14.59	≥10	14.43	0.58	0.64	0.94
≥17	12.74	≥10	14.43	0.57	0.63	0.94
≥18	11.09	≥11	11.62	0.56	0.61	0.95
≥19	9,39	≥12	9.16	0.55	0.59	0.96
≥20	7.92	≥12	9.16	0.54	0.51	0.96
≥21	6.44	≥13	7.13	0.53	0.57	0.97
≥22	5.35	≥14	5.44	0.49	0.51	0.97

GHQ‐12 (bi‐modal scoring) cut‐off values	GHQ12 (bi‐modal scoring) prevalence	K6 cut‐off values	K6 prevalence	Kappa coefficient	Positive agreement	Negative agreement
≥2	27.96	≥7	24.84	0.55	0.67	0.88
≥3	22.05	≥8	20.87	0.56	0.66	0.91
≥4	18.08	≥9	17.17	0.54	0.62	0.92
≥5	14.76	≥10	14.43	0.54	0.61	0.93
≥6	11.88	≥11	11.62	0.53	0.58	0.94
≥7	9.67	≥12	9.16	0.51	0.56	0.95
≥8	7.41	≥13	7.13	0.48	0.52	0.96
≥9	5.79	≥14	5.44	0.45	0.48	0.97

## 4 DISCUSSION

In this study, we found that the GHQ‐12 and the K6 to a large extent measure the same underlying construct, dominated by depressive symptoms. Both scales were found to have the best assessment properties in the unhealthy part of the spectrum (similar target). The conversion tables (Tables 2 and 3) show which specific scores of the K6 equate to scores of the GHQ‐12. These values can be used to monitor prevalence and trends over time, also in studies where one scale has replaced the other. However, it should be noted that those who were identified as cases on one scale were not exactly the same individuals that were identified as cases on the other scale.

GHQ‐12 and K6 differ in number of questions, response options and to some extent content, which means that the scales may function differently. The correlation between the two scales, using the Pearson and Spearman methods, could be classified as just below strong (*r* = 0.77), but above what is often considered to be acceptable for linking (*r* = 0.70) (Fayers & Hays, 2014). The factor analysis indicated that the scales had more in common than they differed, but the GHQ‐12 tended to show a more complex structure. This is in agreement with previous studies on the K6 (Kessler et al., 2010) and GHQ‐12 (Hankins, 2008; Werneke et al., 2000). The differences are likely because the K6 was developed primarily with factor analysis in order to achieve unidimensionality, while the GHQ‐12 was developed with the primary aim to distinguish patients from non‐patients. The dimensionality of the GHQ‐12 has been the focus of several studies. Exploratory factor analyses have suggested one dimension as well as 2‐3 dimensions, but a meta‐analytic study (Gnambs & Staufenbiel, 2018) concluded that GHQ‐12 was essentially unidimensional, with an ECV from Bi‐factor analysis of 79% leaving little variance to subdomains, which is on concert with our ECV of 70% for GHQ‐12 and K6 combined. A Rasch analysis on a longer version of the scale, the GHQ‐30, found that the dimensionality does not necessarily come from the content of the questions, but is a result of the questions having different answer options (Andrich & Van Schoubroeck, 1989). Like our study, that study found that for positively worded items, more response options were used among those low on mental health problems. Thus, the more complex structure of the GHQ‐12 may be due to design (different type of response options), rather than content of the items. Cut‐off recommendations are commonly based on sensitivity and specificity, but while studies often make claims of certain sensitivity and specificity being optimal (based on e.g., Youden's index or highest sum of sensitivity and specificity) this depends on the purpose of the study and the base rate.

The agreement between GHQ‐12 and Kessler when cut offs are calibrated are in line with those found for example, self‐assessed depression scales when compared with diagnostic interviews (Eaton et al., 2007).

### 4.1 Strengths and limitations

A limitation of the study is that the participants were not assessed with a structured psychiatric interview. Psychiatric interviews are generally considered gold standard when determining whether a person has a diagnosis or not and are therefore used to test questionnaires' cut‐offs for diagnoses. Thus, this study cannot answer which specific cut‐off values on the GHQ‐12 or the K6 best corresponds to such case finding. It should be noted that distress, the phenomena targeted, is measured to screen for disorders but may also be used as a continuous measure in its own right, without cut offs. The single group design, specifically the volume of questionnaires administered, may have contributed to rater fatigue. Moreover, the order of the scales was not randomized and the GHQ‐12 was presented last. The potential effect of order could not be evaluated. Another potential limitation is that individuals with severe mental illness (e.g., in inpatient care) may be less likely to respond to surveys and could therefore be less represented in the sample than in the community.

Strengths of the study include the large sample size of 4303 individuals being assessed with parallel tests and the random sampling of individuals in the Stockholm region. The study had a comparably high response rate (47%) which is also a strength. The use of equipercentile equating is yet another strength which enabled comparison of scores even though the relation between the scales was not linear.

## 5 CONCLUSIONS

The GHQ‐12 and K6 are measures of the same phenomena, distress, but with different score distributions. Scale scores of the two can be linked, showing which score best agrees with scores on the other, which is useful for comparisons. Linking the scale scores does not imply total agreement, but the moderate agreement is at par with diagnostic self‐assessment scales.

## AUTHOR CONTRIBUTIONS

**Andreas Lundin**: Conceptualization; formal analysis; investigation; validation; visualization; writing – original draft; writing – review & editing; methodology. **Joseph Junior Muwonge**: Investigation; writing – original draft; writing – review & editing; validation. **Maria Lalouni**: Investigation; writing – original draft; writing – review & editing; validation. **Johan Åhlén**: Conceptualization; investigation; writing – review & editing; project administration.

## CONFLICT OF INTEREST STATEMENT

The authors have no conflicts to report.

## ETHICS STATEMENT

This study received ethical approval (Dnr 2023‐07137‐01), participants in the original project (Hälsa Stockholm 2021) provided written informed consent, and this study adhered to the Helsinki Declaration.

## Supporting information

Supporting Information S1

## Data Availability

The data that support the findings of this study are available on request from the corresponding author. The data are not publicly available due to privacy or ethical restrictions.
