# Supplementary material for: Measuring psychological distress using the 12‐item general health questionnaire and the six‐item Kessler psychological distress scale. Psychometric comparison and equipercentile equating of the two scales
Source: Int J Methods Psychiatr Res. 2024 Jul 4;33(3):e2033. doi: 10.1002/mpr.2033 (PMC11223604; doi:10.1002/mpr.2033)
Supplement: Supplementary file 1 — Supporting Information S1 [file MPR-33-e2033-s001.docx]

**Measuring psychological distress using the 12-item General Health Questionnaire and the six-item Kessler Psychological Distress Scale.** *Psychometric comparison and Equipercentile equating of the two scales.*

Andreas Lundin ^1,2^ ([andreas.lundin@ki.se](mailto:andreas.lundin@ki.se) ; ORCID: 0000-0002-2318-2113), Joseph Jr. Muwonge ^1,2^ ([joseph.junior.muwonge@ki.se](mailto:joseph.junior.muwonge@ki.se) ; ORCID: 0000-0002-9219-9752), Maria Lalouni ^1,3^ ([maria.lalouni@ki.se](mailto:maria.lalouni@ki.se) ; ORCID: 0000-0002-6818-8156), Johan Åhlén ^1,2^  ([johan.ahlen@ki.se](mailto:johan.ahlen@ki.se) ; ORCID: 0000-0003-1415-2200)

1. Centre for Epidemiology and Community Medicine, Stockholm, Sweden
2. Department of Global Public Health, Karolinska Institute, Stockholm, Sweden
3. Department of Clinical Neuroscience, Karolinska Institute, Stockholm, Sweden

Corresponding author

Name: Andreas Lundin

Contact: [andreas.lundin@ki.se](mailto:andreas.lundin@ki.se)

**Supplementary material**
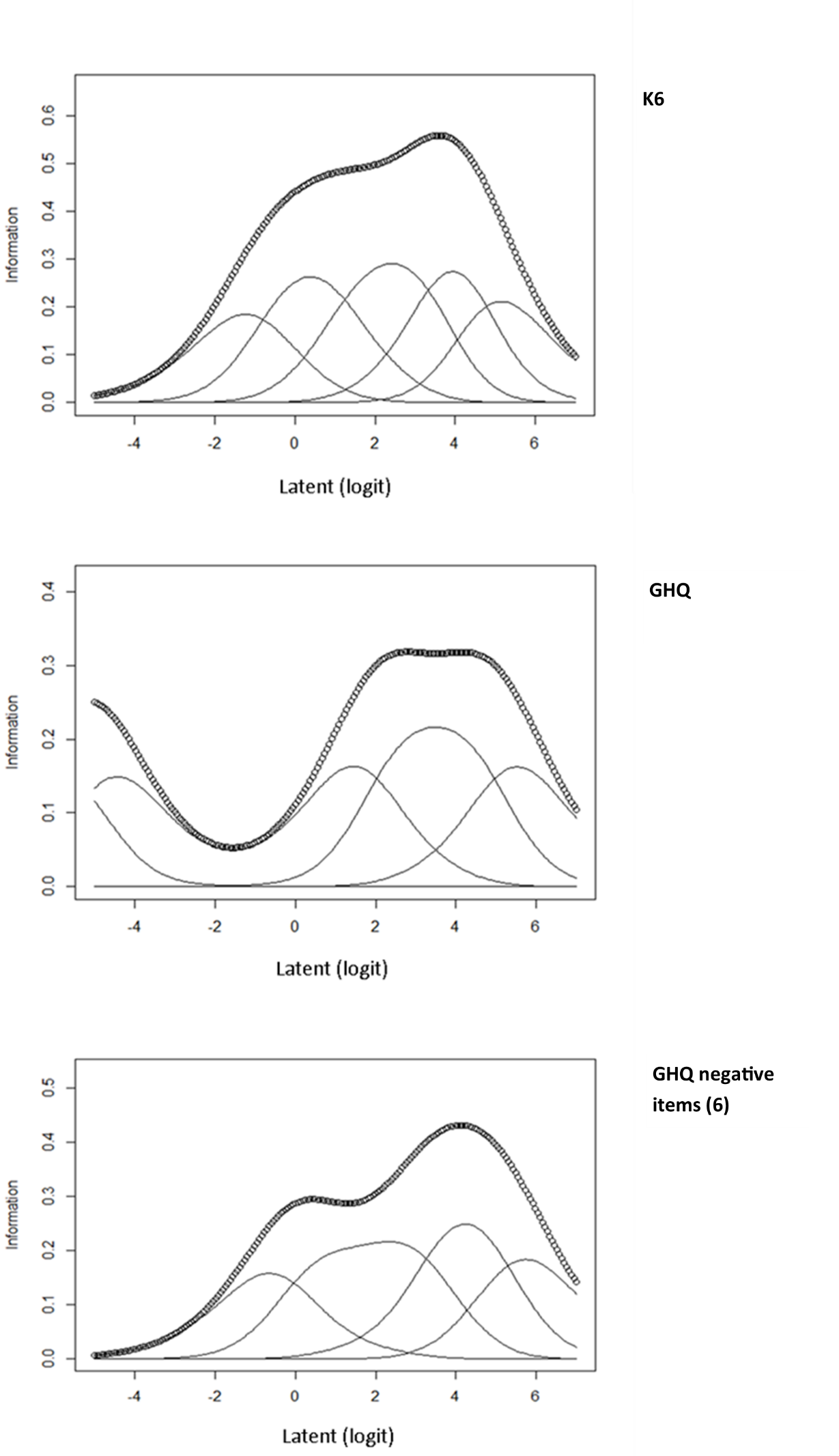


**Figure S1-S3. Information curves, total and for response options**


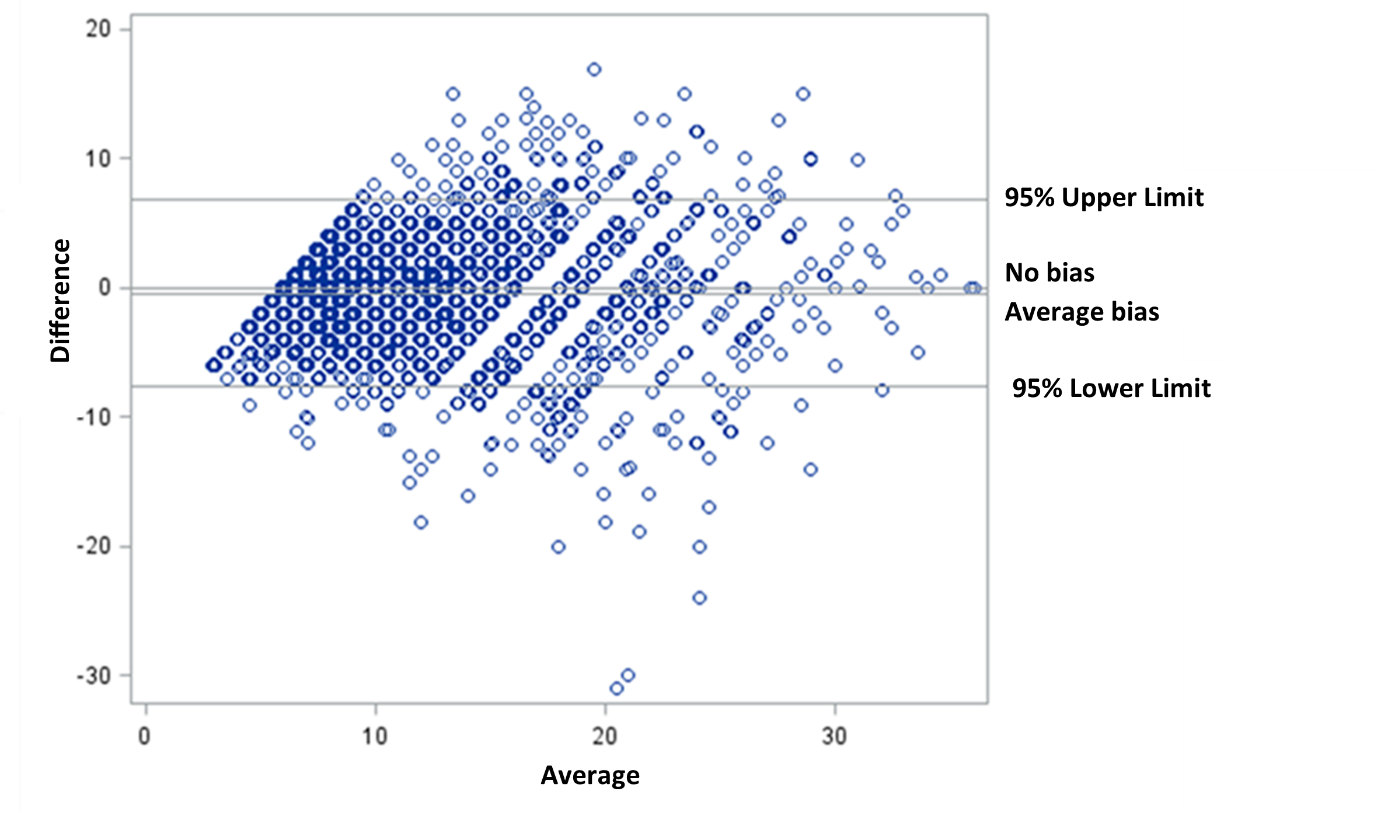


**Figure S4. Bland-Altman plot, average of GHQ-12 and K6 scores equivalent to GHQ-12 (x-axis) and the difference between GHQ-12 and equivalent GHQ-12.**
